# Perception of Radiation Risk as a Predictor of Mid-Term Mental Health after a Nuclear Disaster: The Fukushima Health Management Survey

**DOI:** 10.3390/ijerph14091067

**Published:** 2017-09-15

**Authors:** Itaru Miura, Masato Nagai, Masaharu Maeda, Mayumi Harigane, Senta Fujii, Misari Oe, Hirooki Yabe, Yuriko Suzuki, Hideto Takahashi, Tetsuya Ohira, Seiji Yasumura, Masafumi Abe

**Affiliations:** 1 Department of Neuropsychiatry, School of Medicine, Fukushima Medical University, Fukushima 960-1295, Japan; hyabe@fmu.ac.jp; 2 Radiation Medical Science Center for the Fukushima Health Management Survey, Fukushima 960-1295, Japan; mnagai@fmu.ac.jp (M.N.); masagen@fmu.ac.jp (M.M.); harigane@fmu.ac.jp (M.H.); fuji-sen@fmu.ac.jp (S.F.); htaka@fmu.ac.jp (H.T.); fwge1119@mb.infoweb.ne.jp (T.O.); yasumura@fmu.ac.jp (S.Y.); masafumi@fmu.ac.jp (M.A.); 3 Department of Epidemiology, School of Medicine, Fukushima Medical University, Fukushima 960-1295, Japan; 4 Department of Disaster Psychiatry, School of Medicine, Fukushima Medical University, Fukushima 960-1295, Japan; 5 Department of Neuropsychiatry, School of Medicine, Kurume University, Fukuoka 830-0011, Japan; oe_misari@kurume-u.ac.jp; 6 Department of Adult Mental Health, National Institute of Mental Health, National Center of Neurology and Psychiatry, Tokyo 187-8553, Japan; yrsuzuki@ncnp.go.jp; 7 Department of Public Health, School of Medicine, Fukushima Medical University, Fukushima 960-1295, Japan

**Keywords:** risk perception, nuclear disaster, mid-term mental health, evacuation, predictive factor

## Abstract

Predictive factors including risk perception for mid-term mental health after a nuclear disaster remain unknown. The purpose of this study was to examine the association between perceived radiation risk and other factors at baseline and mid-term mental health after the Fukushima Daiichi nuclear disaster of 2011 in Japan. A mail-based questionnaire survey was conducted in January 2012 and January 2013. Mental health status was assessed using the K6 scale. Psychological distress over the 2-year period was categorized into the following four groups: chronic, recovered, resistant, or worsened. Most participants (80.3%) were resistant to the disaster. A positive association was found between the radiation risk perception regarding immediate effects and the worsened group in women. Baseline post-traumatic stress disorder (PTSD) or a history of psychiatric disease predicted being in the chronic or worsened group in mid-term course. These results suggest that evacuees who believed that their health was substantially affected by the nuclear disaster were at an increased risk of having poor mid-term mental health in women. Careful assessment of risk perception after a nuclear disaster, including the presence of PTSD or a history of psychiatric disease, is needed for appropriate interventions.

## 1. Introduction

Although post-traumatic stress disorder (PTSD) is the most common psychiatric reaction to disaster, depression, which often coexists with PTSD [1], is also important because it may lead to suicidal ideation. Because the prevalence of psychological morbidity including depression and anxiety typically decrease after a disaster [2,3,4], being able to predict the worsening of mental health problems among sufferers is important in order to provide appropriate care and support from the early phase.

Many studies have reported risk factors for mental health after disasters, suggesting that disaster exposure [5,6,7], general neurotic personality [5], aging, female sex, job stress [8], and baseline PTSD [9] predicted poor mid-term mental health. A recent systematic review [10] showed that various factors were associated with mental health status; i.e.; (1) factors related to the disaster (e.g., higher level of exposure; those who were evacuated; those who suffered financial losses; loss of employment), (2) coping factors (e.g., positive and proactive behaviors), (3) health-related factors (e.g., poor mental or physical health status before disaster), (4) personal factors (e.g., female or male gender; older age; lower the socioeconomic status). Moreover, a recent meta-analysis [11] suggested that predictors for depression after disasters included both pre-existing personal factors (female gender, not married; holding religious beliefs; poor education; prior trauma) and disaster-related factors (disaster experience, loss of employment or property, suffering house damage).

After the Chernobyl nuclear power plant accident in 1986, exposure to a nuclear disaster [12], female sex [13], close proximity to the disaster site [14,15], perception of radiation risk [15], and being an evacuee [16,17] were all associated with poor long-term mental health. However, no well-designed longitudinal studies were conducted in the first three years after the accident. The Fukushima Daiichi nuclear disaster resulting from the Great East Japan Earthquake on 11 March 2011, forced many people in Fukushima Prefecture to evacuate from their hometowns. A recent cross-sectional study [18] reported that the perception of radiation risk was associated with psychological distress after the disaster. Risk perception is important as a cognitive factor, and a cognitive model of PTSD [19] suggested that PTSD becomes persistent when individuals process the trauma in a way that leads to a sense of serious, current threat. Previous studies showed that perception of risk was associated with mental health problems such as PTSD, acute stress disorder, and depression after nuclear [15,20,21,22] or non-nuclear [23,24] disaster. Moreover, incorrect understanding of effects of radiation on health was associated with bad mental health status in Nagasaki, Japan [25]. Although risk perception is influenced by disaster experiences [26], it has been suggested that perception of risk may have considerable effects on mental health status, in particular after nuclear accident. Because there are no well-designed longitudinal studies regarding associations between perceived radiation risk and other predictive factors at baseline and mid-term mental health problems after a nuclear disaster, we examined the association between the perception of radiation risk and other predictive factors at baseline and mid-term mental health after the Fukushima Daiichi nuclear disaster using longitudinal data from the large-scale Fukushima Health Management Survey [27].

## 2. Materials and Methods

### 2.1. Participants

The Fukushima Health Management Survey is a mail-based questionnaire survey focusing on lifestyle and mental health among adult residents (aged ≥15 years on 11 March 2011) of the evacuation zone designated by the Japanese government (i.e., Hirono town, Naraha town, Tomioka town, Kawauchi village, Okuma town, Futaba town, Namie town, Katsurao village, Minamisoma city, Tamura city, Kawamata town, Iitate village, and part of Date city in Fukushima Prefecture) [27,28] that was first carried out in fiscal year (FY) 2011 (January 2012, 10 months after the disaster). A follow-up survey was conducted in FY2012 (January 2013). There were 180,604 targeted residents of the evacuation zone in FY2011 and 184,507 in FY2012. Figure S1 shows a flowchart of the study selection and inclusion procedures. No responses were received from 107,171 and 32,220 residents in FY2011 and FY2012, respectively. In addition, 5157 respondents were excluded from analysis because of missing data. Finally, 36,056 participants were included in the analysis.

### 2.2. Measures

The outcome of this study was scores on the K6 scale [29], which is composed of six questions (score range: 0–24) that assess non-specific psychological distress during the previous 30 days. The Japanese version of the K6 scale has been validated [30]. In this study, we adopted the cut-off score of ≥13, which is classified as indicating probable severe mental illness [31]. The PTSD Checklist Stressor-Specific Version (PCL-S) [32] was used to assess post-traumatic symptoms. The PCL-S is a 17-item self-report measure, and the Japanese version has been shown to have sufficient validity and reliability [33]. The cut-off was set as a PCL-S score of ≥44 [32]. Data was collected on the participants’ sociodemographic characteristics, including age, sex, education, and living status, as well as subjective health status, perceived radiation risk, and disaster experiences and history. With regard to perceived radiation risk, we asked participants about their beliefs on the potential risks of radiation exposure [34] using the following questions: (i) “What is the likelihood of suffering immediate health damage (e.g., dying within 1 month) as a result of your current level of radiation exposure?”; (ii) What is the likelihood of damage to your health (e.g., cancer onset) in later life as a result of your current level of radiation exposure?”; and (iii) What is the likelihood that the health of your future (i.e., as of yet unborn) children and grandchildren will be affected as a result of your current level of radiation exposure?” Participants were asked to respond to each question using a 4-point Likert scale (1, very unlikely; 2, unlikely; 3, likely; 4, very likely). These items were translated into Japanese, then back to English, and modified after discussion with the authors of the questionnaire. We sent reminders to non-responders to the survey, but there were no incentives for the participants. This study was approved by the ethics committee of Fukushima Medical University (No. 1316). We obtained written informed consent from guardians on behalf of the children enrolled in the study to conduct an epidemiologic study based on the guidelines of the Council for International Organizations of Medical Science [35]. The study was conducted in accordance with the approved guidelines.

### 2.3. Statistical Analysis

Only the participants who responded to the K6 scale in both FY2011 and FY2012 were included in the analysis. Based on K6 scores from both years, we divided mental health status over the 2-year period into the following four groups: chronic group (K6 score ≥ 13 in both FY2011 and FY2012); recovered group (K6 score ≥ 13 in FY2011 and <13 in FY2012); resistant group (K6 score < 13 in both FY2011 and FY2012); and worsened group (K6 score < 13 in FY2011 and ≥13 in FY2012). To investigate predictive factors for mid-term mental health status, we focused the changes in psychological distress (K6) from baseline. We used logistic regression analysis to investigate the predictive factors at baseline associated with: (1) worsened group (compared with resistant group; K6 score < 13 at baseline in both groups) and (2) recovered group (compared with chronic group; K6 score ≥ 13 at baseline in both groups). Multivariate logistic regression analysis adjusting all predictors (age, subjective health status, past history, disaster experiences, living place and arrangement in 2011, posttraumatic stress symptoms in 2011, and radiation risk perception in 2011 (immediate, delayed, and genetic effects)) were used. We analyzed males and females separately because gender may have effects on mental health status after disaster [11,36,37]. All statistical analyses were conducted using SAS (version 9.3; SAS Institute Inc., Cary, NC, USA).

## 3. Results

### 3.1. Participant Characteristics

The baseline characteristics of the four groups are shown in Table 1. For the total population, the mean scores (±standard deviation) on the K6 scale were 6.2 ± 5.7 in FY2011 and 5.7 ± 5.5 in FY2012.

The prevalence of being at high risk for psychological distress (K6 score ≥ 13) was 14.3% in FY2011 and 11.7% in FY2012, which are relatively high compared with the data of non-disaster settings in Japan (4.7%, among 15–64 years old) [38]. In those who responded in only FY2011 (and did not respond in FY2012), the prevalence of K6 score ≥ 13 was 15.2% in FY2011, and was slightly higher than those who respond in both FY2011 and FY 2012. The whole sample was categorized according to the definition stated above as follows: chronic group (6.5%); recovered group (7.8%); resistant group (80.3%); and worsened group (5.3%).

### 3.2. Predictors for The Worsened Group Compared with the Resistant Group

Table 2 shows the results of univariate and multivariate analyses of potential factors in the worsened group compared with the resistant group (the results in total subjects are shown in Table S1). In univariate analysis, the perception that immediate (odds ratio [OR]: 2.82, 95% confidence interval [CI]: 2.18–3.64 in men; OR: 2.97, 95% CI: 2.36–3.73 in women), delayed (OR: 2.96, 95% CI: 2.38–3.67 in men; OR: 2.31, 95% CI: 1.90–2.80 in women), or genetic (OR: 2.78, 95% CI: 2.19–3.52 in men; OR: 2.33, 95% CI: 1.88–2.89 in women) radiation effects were very likely was positively associated with the worsened group (Table 2). Similarly, the perception that immediate (OR: 2.33, 95% CI: 1.82–2.99 in men; OR: 2.20, 95% CI: 1.79–2.70 in women), delayed (OR: 2.04, 95% CI: 1.62–2.57 in men; OR: 1.82, 95% CI: 1.50–2.22 in women), or genetic (OR: 1.70, 95% CI: 1.32–2.20 in men; OR: 1.51, 95% CI: 1.21–1.89 in women) effects were likely was positively associated with the worsened group (Table 2). In multivariate analysis, after controlling for individual and disaster-related factors, the significant associations for perceived radiation risk regarding immediate effects remained significant among women in the worsened group. On the other hand, having bad or very bad subjective health status, a history of psychiatric disease, a PCL-S score ≥ 44, a history of cardiovascular disease among women, the experience of having heard the sound of the nuclear plant explosion among women, the experience of partial collapse or more severe damage to one’s own home among women, and living in a dwelling other than one’s own home were positively associated with the worsened group, whereas age ≥40 years among men was negatively associated with the worsened group (Table 2).

### 3.3. Predictors for the Recovered Group Compared with the Chronic Group

Results from univariate and multivariate analyses of potential factors for the recovered group compared with the chronic group are shown in Table 3 (the results in total subjects are shown in Table S1). In univariate analysis, the perceived risk that immediate (OR: 0.75, 95% CI: 0.57–0.97), delayed (OR: 0.72, 95% CI: 0.53–0.98), or genetic (OR: 0.66, 95% CI: 0.46–0.94) effects were very likely were negatively associated with the recovered group among men (Table 2).

Among women, the perceived risk that immediate effects were likely (OR: 0.76, 95% CI: 0.61–0.94) or very likely (OR: 0.74, 95% CI: 0.60–0.92) was negatively associated with the recovered group (Table 3). In multivariate analysis, after controlling for individual and disaster-related factors, the significant associations between perceived radiation risk and the recovered group became non-significant. On the other hand, having usual or better subjective health status, a PCL-S score < 44, previous experience with tsunamis among women, living in Fukushima Prefecture among men, and living in one’s own home among women were positively associated with the recovered group, whereas a history of psychiatric disease was negatively associated with the recovered group (Table 3).

## 4. Discussion

To the best of our knowledge, this is the first longitudinal study conducted to investigate the association between perceived radiation risk at baseline and mid-term mental health after a nuclear accident in a large sample. In this study, most (80.3%) of the 36,056 evacuees were resistant to the disaster. This is consistent with a previous finding that few people develop chronic mental disorders after traumatic events [39]. Our results suggest that most sufferers are resistant to the disaster, even if disaster is complex, including an earthquake, tsunami, and nuclear accident. Notably, a greater proportion of the respondents were classified as being in the recovered group (7.8%) compared with the worsened group (5.3%). While the chronic stress resulting from the complex disaster worsened the mental health status of some of the participants, more showed resilience. Therefore, this study investigated predictive factors for both the recovered and the worsened groups.

We also found that the degree of perceived radiation risk predicted the degree of worsening mental health status after the disaster, although multivariate analysis revealed that the association between perceived radiation risk regarding immediate effects and the worsened group remained significant among only women after controlling for individual and disaster-related factors. This finding was consistent with that from a recent cross-sectional study demonstrating that perceived radiation risk was associated with psychological distress after the Fukushima disaster [18]. Previous studies investigating mental health after the Chernobyl nuclear accident found that poor mental health status could be explained by risk perception [20,21,22]; however, those studies were conducted at least 6.5 years after the nuclear accident. Interestingly, our results of the association between perceived radiation risk and the worsened group was observed among only women with regard to immediate effects after multivariate analysis. A recent meta-analysis demonstrated that being female was significantly associated with depression after natural disaster [11]. Furthermore, many studies suggested that women have greater tendency to express emotions such as fear after traumatic events [40,41], and were less likely to use positive coping strategies [36]. Taken together, emotionality and coping strategies of women may account for our results of the association between perceived radiation risk regarding immediate effects and the worsened group among women. On the other hand, women might have more sensitive biological stress systems such as hypothalamic-pituitary-adrenal axis, which might have effects on the results. Boe et al. [5] investigated the association between pre-disaster vulnerability and increased risk of psychopathology, and found that a general neurotic personality predicted chronic psychopathology after a disaster. Although risk perception is influenced by disaster experiences [26], having a vulnerable personality may also affect the perception of radiation risk. Our results indicated that a perception of high radiation risk predicted worse psychological well-being after the disaster; this finding suggests that evacuees who believe that their health is highly affected by a nuclear accident are at a high risk of late-onset severe mental illness such as depression or anxiety disorder. Conversely, depressive symptoms among evacuees might influence their risk perception due to alternations in their cognitive processes, which is likely to be a vicious circle. Early detection and careful intervention are needed to prevent a deterioration of mental health among evacuees who believe that their health is highly affected by radiation. In particular, population scale intervention based on cognitive models of PTSD [19] may be helpful to reduce risk perception among vulnerable groups [42]. Although risk communication is challenging, it is necessary in order to help evacuees make well-informed decisions about life changes [43].

Having PTSD symptoms related to the nuclear disaster at baseline was significantly associated with mental health status during the 2-year period of this study; this finding was consistent with those from previous study [9] reporting that the presence of PTSD symptoms at baseline predicted poor mid-term mental health. Moreover, it is well known that depression often coexists with PTSD [1]. Similarly, a history of psychiatric disease was strong predictor for psychological distress. Although we did not assess the diagnosis of the psychiatric disease, it is estimated that patients with neurotic disorder might have fear and anxiety concerning disasters, which leads to persistent anticipatory anxiety [44]. Those who have a psychiatric disease have more vulnerability to distress [4], and were more strongly affected by forced evacuations and other similar experiences after the Fukushima disaster, which was a complex disaster involving a nuclear accident. Taken together, the coexistence of PTSD or past psychiatric disease were strong predictors of poor mid-term mental health. Since complex disasters have various effects on the lives of evacuees, including secondary effects such as new living arrangements or changes in job status, the presence of trauma and vulnerability to distress should be assessed from the early phase after a disaster.

Our results regarding the positive association between the worsened group and not living in one’s own home are consistent with those from a previous systematic review [45] reporting that relocated individuals were more likely to experience psychological distress or depression after a disaster. Interestingly, living in Fukushima Prefecture in 2011 was a significant predictor among men in the recovered group, whereas no such association was observed among women. Although no association was found between living outside of Fukushima Prefecture and psychological distress at baseline [18], living outside of Fukushima Prefecture may cause various changes in daily life, such as in job or financial status, especially among men. Our results suggest that place of residence may affect mid-term psychological well-being, and thus outreach services are needed to provide careful intervention not only inside, but also outside the prefecture.

This study did have several limitations. First, a relatively low proportion of the target population was included in the analysis, which might have affected the interpretation of the results. Previous studies [46,47] showed that the mental health status might have effects on response rate to survey, suggesting that non-response was associated with bad mental health status. There might be many residents who were in a bad condition and could not answer the survey. This is important limitation of this study. Second, insufficient information was available regarding pre-disaster factors such as personality traits, social adaptation traumatic experiences, or other mental health problems including addictive behavior. Third, the several risk factors in this study are general risk factors, and may be related to general mental health rather than disaster-specific problems. Finally, the baseline survey was conducted 10 months after the disaster, which is a relatively long time because the baseline might include subsequent changes that do not reflect the status of the evacuees just after the disaster. Additionally, we did not assess whether the subjects received psychological interventions. Nevertheless, to our knowledge, this was the first longitudinal study on the association between perceived radiation risk and mid-term mental health after a nuclear accident. Our finding that a perception of high radiation risk is a predictor for worse mid-term mental health in women is expected to contribute to the assessment of and interventions for evacuees after nuclear accidents.

## 5. Conclusions

In summary, we found a significant association between perceived radiation risk regarding immediate effects and mid-term mental health after a nuclear accident in women, indicating that female evacuees who believe that their health is highly affected by a nuclear accident are at an increased risk of poor mid-term mental health. Although it is difficult to generalize these findings to other disasters, the assessment of perceived risk and other predictors such as PTSD symptoms or a history of psychiatric disease is needed in order to promote careful intervention for affected populations.

## Figures and Tables

**Table 1:** Baseline characteristics of each group in 2011.

	Chronic	Recovered	Resistant	Worsened
	(K6 ≥ 13 ⇒ K6 ≥ 13)	(K6 ≥ 13 ⇒ K6 < 13)	(K6 < 13 ⇒ K6 < 13)	(K6 < 13 ⇒ K6 ≥ 13)
**Men**				
No. of participants	790	995	12,911	757
Mean age (years)	57.7 ± 16.4	58.0 ± 16.4	57.9 ± 16.9	57.9 ± 17.6
15–39 years (%)	17.0	17.4	17.6	19.7
40–64 years (%)	45.4	44.0	43.8	39.0
≥65 years (%)	37.6	38.6	38.6	41.4
Subjective health status				
bad or very bad (%)	60.7	44.9	12.4	32.4
Disaster experiences				
Tsunami (%)	31.4	31.5	23.6	28.1
Heard the sound of nuclear plant explosion (%)	70.6	68.7	55.7	66.6
Bereavement				
Yes (%)	30.3	28.5	18.4	23.5
House damage				
No damage (%)	16.2	17.6	26.9	21.3
Partial collapse (%)	66.6	69.5	65.3	68.6
Half collapse and worse (%)	17.3	12.9	7.9	10.1
Past history				
Cardiovascular disease (heart disease and stroke) (%)	23.0	22.4	14.5	18.0
Psychiatric disease (%)	20.8	12.9	2.8	8.3
Living place				
Out of Fukushima prefecture (%)	25.8	20.9	16.7	22.6
Living arrangement				
Other than own home (%)	80.1	76.9	64.1	77.0
Posttraumatic stress symptoms				
PCL-S score ≥ 44 (%)	81.0	68.8	8.9	36.2
Radiation Risk Perception				
Immediate effect				
Very unlikely (%)	43.4	48.0	71.9	58.0
Unlikely (%)	23.7	22.5	17.9	21.0
Likely (%)	14.3	14.2	5.7	10.8
Very likely (%)	18.6	15.3	4.5	10.2
Delayed effect				
Very unlikely (%)	11.0	13.8	29.7	17.7
Unlikely (%)	18.7	20.3	32.2	25.9
Likely (%)	24.2	24.4	19.7	23.9
Very likely (%)	46.1	41.5	18.5	32.5
Genetic effect				
Very unlikely (%)	7.1	9.5	20.8	12.1
Unlikely (%)	12.2	14.1	27.8	20.0
Likely (%)	20.2	23.0	24.0	23.8
Very likely (%)	60.5	53.4	27.3	44.2
**Women**				
No. of participants	1464	1748	15,183	1091
Mean age (years)	58.6 ± 17.6	57.1 ± 17.4	54.5 ± 17.9	57.4 ± 18.5
15–39 years (%)	18.0	19.8	25.2	21.5
40–64 years (%)	42.7	44.2	43.5	40.2
≥65 years (%)	39.3	36.0	31.3	38.4
Subjective health status				
bad or very bad (%)	61.7	43.3	12.1	33.8
Disaster experiences				
Tsunami (%)	22.5	24.7	16.4	21.6
Heard the sound of nuclear plant explosion (%)	69.3	66.5	48.8	63.0
Bereavement				
Yes (%)	33.3	30.3	18.6	24.3
House damage				
No damage (%)	19.6	20.0	30.4	20.6
Partial collapse (%)	66.7	68.7	61.6	66.4
Half collapse and worse (%)	13.8	11.3	8.0	13.0
Past history				
Cardiovascular disease (heart disease and stroke) (%)	18.3	14.1	8.1	15.8
Psychiatric disease (%)	24.8	12.7	3.3	11.4
Living place				
Out of Fukushima prefecture (%)	25.3	23.3	20.9	23.0
Living arrangement				
Other than own home (%)	81.8	76.3	66.7	76.3
Posttraumatic stress symptoms				
PCL-S score ≥ 44 (%)	83.7	71.9	11.0	39.3
Radiation Risk Perception				
Immediate effect				
Very unlikely (%)	46.6	50.7	70.6	55.7
Unlikely (%)	22.9	24.4	19.0	23.7
Likely (%)	15.4	12.7	6.6	11.4
Very likely (%)	15.1	12.2	3.9	9.2
Delayed effect				
Very unlikely (%)	9.5	10.4	22.5	14.9
Unlikely (%)	18.5	22.2	33.0	24.6
Likely (%)	26.2	26.6	24.2	29.3
Very likely (%)	45.8	40.9	20.4	31.2
Genetic effect				
Very unlikely (%)	5.2	5.6	15.0	10.0
Unlikely (%)	11.5	13.7	28.0	16.4
Likely (%)	21.7	26.3	28.0	28.3
Very likely (%)	61.6	54.4	29.1	45.3

PCL-S, Post-Traumatic Checklist Stressor-Specific Version.

**Table 2:** Univariate and multivariate analysis by logistic regression comparing worsened with resistant group.

		Worsened (K6 < 13 ⇒ K6 ≥ 13) (Reference: Resistant; K6 < 13 ⇒ K6 < 13)							
		Univariate Analysis				Multivariate Analysis			
		Men		Women		Men		Women	
		OR [95% CI]	*p* Value	OR [95% CI]	*p* Value	OR [95% CI]	*p* Value	OR [95% CI]	*p* Value
Age groups	15–39 years	Reference		Reference		Reference		Reference	
	40–64 years	0.80 (0.65–0.97)	0.008	1.09 (0.92–1.28)	0.122	0.70 (0.56–0.89)	0.003	0.84 (0.69–1.02)	0.072
	≥65 years	0.96 (0.78–1.17)	0.365	1.44 (1.22–1.70)	<0.001	0.75 (0.59–0.97)	0.025	0.82 (0.67–1.02)	0.068
Subjective health status	Usual, good, or very good	Reference		Reference		Reference		Reference	
	Bad or very bad	3.39 (2.88–3.99)	<0.001	3.69 (3.22–4.23)	<0.001	2.27 (1.87–2.76)	<0.001	2.38 (2.01–2.82)	<0.001
Past history	Cardiovascular disease (heart disease and stroke)	1.29 (1.07–1.56)	0.009	2.12 (1.79–2.52)	<0.001	1.07 (0.85–1.35)	0.551	1.45 (1.16–1.81)	0.001
	Psychiatric disease	3.19 (2.40–4.23)	<0.001	3.73 (3.02–4.61)	<0.001	1.92 (1.38–2.67)	<0.001	2.58 (2.02–3.30)	<0.001
Disaster experiences	Tsunami	1.27 (1.08–1.49)	0.004	1.41 (1.21–1.64)	<0.001	1.00 (0.82–1.22)	1	0.95 (0.78–1.15)	0.576
	heard the sound of nuclear plant explosion	1.59 (1.36–1.85)	<0.001	1.79 (1.57–2.03)	<0.001	1.13 (0.94–1.36)	0.194	1.26 (1.08–1.46)	0.004
	bereavement	1.37 (1.15–1.63)	<0.001	1.40 (1.21–1.62)	<0.001	1.05 (0.85–1.28)	0.662	1.01 (0.85–1.20)	0.936
	house damage								
	No damage	Reference		Reference		Reference		Reference	
	Partial collapse	1.33 (1.10–1.60)	0.632	1.60 (1.36–1.87)	0.665	1.11 (0.90–1.37)	0.312	1.26 (1.05–1.50)	0.012
	Half collapse and worse	1.62 (1.21–2.17)	0.01	2.40 (1.91–3.01)	<0.001	0.97 (0.69–1.37)	0.86	1.50 (1.14–1.97)	0.004
Living place in 2011	In Fukushima prefecture	Reference		Reference		Reference		Reference	
	Out of Fukushima prefecture	1.46 (1.22–1.74)	<0.001	1.13 (0.980–1.31)	0.092	1.22 (0.99–1.50)	0.064	0.96 (0.80–1.14)	0.616
Living arrangement in 2011	Own home	Reference		Reference		Reference		Reference	
	Other than own home	1.88 (1.58–2.23)	<0.001	1.60 (1.39–1.85)	<0.001	1.44 (1.18–1.77)	<0.001	1.24 (1.04–1.48)	0.014
Posttraumatic stress symptoms	PCL-S score ≥ 44 in 2011	5.84 (4.97–6.86)	<0.001	5.26 (4.61–6.02)	<0.001	4.04 (3.32–4.90)	<0.001	3.35 (2.85–3.95)	<0.001
Radiation Risk Perception in 2011	Immediate effect								
	Very unlikely	Reference		Reference		Reference		Reference	
	Unlikely	1.46 (1.21–1.76)	0.013	1.58 (1.36–1.84)	0.042	0.98 (0.78–1.22)	0.839	1.32 (1.10–1.58)	0.003
	Likely	2.33 (1.82–2.99)	0.003	2.20 (1.79–2.70)	0.01	1.14 (0.84–1.55)	0.41	1.34 (1.03–1.74)	0.028
	Very likely	2.82 (2.18–3.64)	<0.001	2.97 (2.36–3.73)	<0.001	1.02 (0.72–1.44)	0.921	1.50 (1.10–2.03)	0.01
	Delayed effect								
	Very unlikely	Reference		Reference		Reference		Reference	
	Unlikely	1.35 (1.08–1.69)	<0.001	1.12 (0.92–1.37)	<0.001	1.08 (0.79–1.46)	0.643	1.00 (0.76–1.33)	0.978
	Likely	2.04 (1.62–2.57)	0.005	1.82 (1.50–2.22)	<0.001	1.33 (0.94–1.88)	0.105	1.04 (0.76–1.41)	0.817
	Very likely	2.96 (2.38–3.67)	<0.001	2.31 (1.90–2.80)	<0.001	1.32 (0.90–1.92)	0.155	0.95 (0.68–1.32)	0.755
	Genetic effect								
	Very unlikely	Reference		Reference		Reference		Reference	
	Unlikely	1.23 (0.95–1.61)	0.001	0.88 (0.69–1.12)	<0.001	1.11 (0.78–1.56)	0.568	0.73 (0.53–1.01)	0.055
	Likely	1.70 (1.32–2.20)	0.18	1.51 (1.21–1.89)	0.019	1.08 (0.75–1.57)	0.681	1.04 (0.75–1.44)	0.828
	Very likely	2.78 (2.19–3.52)	<0.001	2.33 (1.88–2.89)	<0.001	1.31 (0.89–1.92)	0.169	1.25 (0.89–1.76)	0.202

OR, odds ratio. OR in multivariate analyses were adjusted for all other variables in table.

**Table 3:** Univariate and multivariate analysis by logistic regression comparing recovered with chronic group.

		Recovered (K6 ≥ 13 ⇒ K6 < 13) (Reference: Chronic; K6 ≥ 13 ⇒ K6 ≥ 13)							
		Univariate Analysis				Multivariate Analysis			
		Men		Women		Men		Women	
		OR [95% CI]	*p* Value	OR [95% CI]	*p* Value	OR [95% CI]	*p* Value	OR [95% CI]	*p* Value
Age groups	15–39 years	Reference		Reference		Reference		Reference	
	40–64 years	0.95 (0.73–1.23)	0.564	0.94 (0.78–1.14)	0.659	1.08 (0.80–1.48)	0.614	0.87 (0.70–1.09)	0.229
	≥65 years	1.00 (0.76–1.31)	0.772	0.84 (0.69–1.02)	0.045	1.15 (0.82–1.60)	0.426	0.85 (0.66–1.08)	0.185
Subjective health status	Bad or very bad	Reference		Reference		Reference		Reference	
	Usual, good, or very good	1.90 (1.57–2.30)	<0.001	2.11 (1.83–2.43)	<0.001	1.70 (1.36–2.13)	<0.001	1.61 (1.36–1.91)	<0.001
Past history	Cardiovascular disease (heart disease and stroke)	0.97 (0.77–1.21)	0.754	0.73 (0.61–0.89)	0.001	1.08 (0.82–1.41)	0.59	0.91 (0.72–1.16)	0.454
	Psychiatric disease	0.56 (0.44–0.73)	<0.001	0.44 (0.37–0.53)	<0.001	0.65 (0.48–0.87)	0.003	0.49 (0.39–0.61)	<0.001
Disaster experiences	Tsunami	1.00 (0.82–1.23)	0.977	1.13 (0.96–1.33)	0.147	1.15 (0.91–1.46)	0.254	1.29 (1.05–1.58)	0.015
	heard the sound of nuclear plant explosion	0.92 (0.75–1.12)	0.39	0.88 (0.76–1.02)	0.091	0.91 (0.72–1.15)	0.436	0.98 (0.82–1.17)	0.794
	bereavement	0.92 (0.75–1.13)	0.43	0.87 (0.75–1.012)	0.072	1.00 (0.79–1.27)	0.997	1.00 (0.83–1.19)	0.97
	house damage								
	No damage	Reference		Reference		Reference		Reference	
	Partial collapse	0.96 (0.74–1.25)	0.17	1.01 (0.84–1.22)	0.142	1.03 (0.77–1.38)	0.825	1.11 (0.90–1.36)	0.342
	Half collapse and worse	0.69 (0.49–0.97)	0.014	0.81 (0.62–1.05)	0.055	0.80 (0.54–1.19)	0.265	0.93 (0.69–1.27)	0.657
Living place in 2011	Out of Fukushima prefecture	Reference		Reference		Reference		Reference	
	In Fukushima prefecture	1.32 (1.06–1.64)	0.015	1.12 (0.95–1.31)	0.187	1.45 (1.11–1.89)	0.006	0.97 (0.80–1.18)	0.773
Living arrangement in 2011	Other than own home	Reference		Reference		Reference		Reference	
	Own home	1.21 (0.96–1.52)	0.099	1.40 (1.18–1.66)	<0.001	1.03 (0.78–1.35)	0.849	1.29 (1.05–1.59)	0.016
Posttraumatic stress symptoms	PCL-S score < 44 in 2011	1.94 (1.55–2.42)	<0.001	2.02 (1.69–2.40)	<0.001	1.65 (1.27–2.15)	<0.001	1.64 (1.33–2.01)	<0.001
Radiation Risk Perception in 2011	Immediate effect								
	Very unlikely	Reference		Reference		Reference		Reference	
	Unlikely	0.86 (0.68–1.09)	0.883	0.98 (0.82–1.17)	0.05	0.99 (0.75–1.31)	0.93	1.03 (0.84–1.27)	0.777
	Likely	0.89 (0.67–1.19)	0.789	0.76 (0.61–0.94)	0.104	1.12 (0.79–1.60)	0.529	0.79 (0.61–1.03)	0.085
	Very likely	0.75 (0.57–0.97)	0.113	0.74 (0.60–0.92)	0.057	0.94 (0.66–1.33)	0.713	0.90 (0.67–1.19)	0.447
	Delayed effect								
	Very unlikely	Reference		Reference		Reference		Reference	
	Unlikely	0.87 (0.62–1.22)	0.745	1.09 (0.84–1.44)	0.049	0.88 (0.55–1.39)	0.579	1.17 (0.82–1.69)	0.39
	Likely	0.81 (0.58–1.12)	0.596	0.93 (0.72–1.20)	0.664	0.82 (0.50–1.34)	0.426	1.02 (0.69–1.49)	0.938
	Very likely	0.72 (0.53–0.98)	0.034	0.82 (0.64–1.04)	0.005	0.93 (0.55–1.56)	0.781	1.06 (0.71–1.57)	0.784
	Genetic effect								
	Very unlikely	Reference		Reference		Reference		Reference	
	Unlikely	0.86 (0.57–1.31)	0.761	1.11 (0.78–1.59)	0.272	0.90 (0.53–1.54)	0.697	0.93 (0.59–1.48)	0.765
	Likely	0.85 (0.58–1.25)	0.834	1.13 (0.81–1.58)	0.109	1.09 (0.63–1.87)	0.768	1.10 (0.69–1.76)	0.686
	Very likely	0.66 (0.46–0.94)	0.002	0.83 (0.60–1.13)	<0.001	0.83 (0.48–1.45)	0.519	0.97 (0.61–1.55)	0.907

## Supplementary Materials

The following are available online at www.mdpi.com/1660-4601/14/9/1067/s1, Figure S1: Flow chart of the study selection, Table S1: Univariate and multivariate analysis by logistic regression in total subjects.
