# Supplementary material for: Perception of Radiation Risk as a Predictor of Mid-Term Mental Health after a Nuclear Disaster: The Fukushima Health Management Survey
Source: Int J Environ Res Public Health. 2017 Sep 15;14(9):1067. doi: 10.3390/ijerph14091067 (PMC5615604; doi:10.3390/ijerph14091067)
Supplement: Supplementary file 1 [file ijerph-14-01067-s001.pdf]

Figure S1. Flow chart of the study selection.

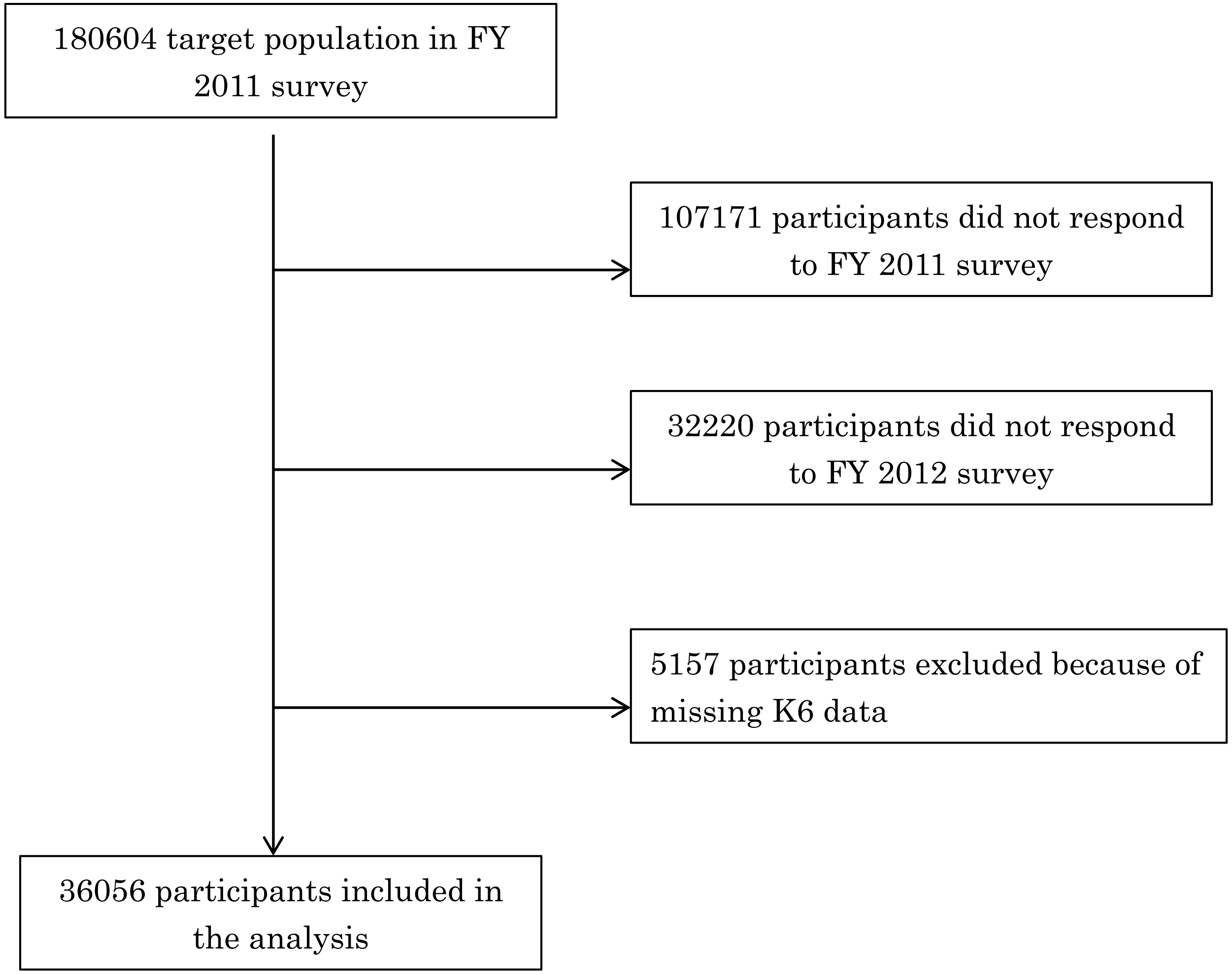

Table S1. Univariate and multivariate analysis by logistic regression in total subjects

		worsened (K6<13⇒K6≥13) (Reference: resistant; K6<13⇒K6<13)		recovered (K6≥13⇒K6<13) (Reference: chronic; K6≥13⇒K6≥13)	
		Univariate analysis	Multivariate analysis	Univariate analysis	Multivariate analysis
		OR [95% CI]	OR [95% CI]	OR [95% CI]	OR [95% CI]
Age groups	15-39 years	Reference	Reference	Reference	Reference
	40-64 years	0.93(0.81-1.07)	0.79(0.68-0.91)	0.96(0.81-1.14)	0.95(0.79-1.14)
	≥65 years	1.14(0.99-1.31)	0.81(0.69-0.95)	0.91(0.77-1.09)	0.95(0.78-1.15)
Subjective health status	Usual, good, or very good	Reference	Reference	1.96(1.73-2.23)	1.64(1.43-1.87)
	Bad or very bad	3.68(3.28-4.12)	2.34(2.06-2.66)	Reference	Reference
Past histroy	Cardiovascular disease (heart disease and stroke)	1.61(1.40-1.86)	1.22(1.04-1.43)	0.85(0.72-0.99)	0.99(0.83-1.18)
	Psychiatric disease	3.82(3.19-4.57)	2.31(1.90-2.81)	0.47(0.39-0.55)	0.54(0.46-0.65)
Disaster experiences	Tsunami	1.33(1.18-1.50)	1.19(1.04-1.36)	1.09(0.95-1.26)	1.23(1.05-1.43)
	heard the sound of nuclear plant explosion	1.69(1.52-1.88)	1.20(1.06-1.35)	0.88(0.77-1.01)	0.95(0.83-1.10)
	bereavement	1.41(1.25-1.60)	1.03(0.90-1.18)	0.93(0.81-1.07)	0.99(0.86-1.15)
	house damage				

	No damage	Reference	Reference	Reference	Reference
	Partial collapse	1.49(1.31-1.69)	1.19(1.04-1.36)	1.02(0.87-1.20)	1.08(0.91-1.28)
	Half collapse and worse	1.98(1.63-2.39)	1.26(1.03-1.56)	0.81(0.65-1.01)	0.88(0.69-1.12)
Living place in 2011	In Fukushima prefecture	Reference	Reference	1.20(1.04-1.40)	1.11(0.95-1.30)
	Out of Fukushima prefecture	1.28(1.13-1.45)	1.06(0.92-1.21)	Reference	Reference
Living arrangement in 2011	Own home	Reference	Reference	1.34(1.15-1.56)	1.19(1.01-1.41)
	Other than own home	1.72(1.52-1.93)	1.33(1.17-1.52)	Reference	Reference
Posttraumatic stress symptoms	PCL-S score ≥44 in 2011	5.65(5.05-6.32)	3.63(3.21-4.12)	Reference	Reference
	PCL-S score <44 in 2011	Reference	Reference	1.90(1.64-2.21)	1.62(1.38-1.90)
Radiation Risk Perception in 2011	Immediate effect				
	Very unlikely	Reference	Reference	Reference	Reference
	Unlikely	1.62(1.43-1.84)	1.17(1.02-1.35)	0.93(0.80-1.09)	1.01(0.86-1.20)
	Likely	2.26(1.90-2.69)	1.25(1.03-1.53)	0.80(0.66-0.97)	0.89(0.72-1.10)
	Very likely	2.77(2.29-3.36)	1.25(0.99-1.57)	0.76(0.63-0.91)	0.91(0.73-1.13)
	Delayed effect				
	Very unlikely	Reference	Reference	Reference	Reference
	Unlikely	1.23(1.05-1.45)	1.05(0.85-1.28)	1.06(0.84-1.34)	1.06(0.80-1.41)
	Likely	1.92(1.63-2.26)	1.16(0.93-1.46)	0.88(0.70-1.11)	0.94(0.70-1.27)
	Very likely	2.59(2.21-3.03)	1.11(0.86-1.42)	0.80(0.65-0.99)	1.01(0.74-1.39)

Genetic effect				
Very unlikely	Reference	Reference	Reference	Reference
Unlikely	1.05(0.87-1.28)	0.89(0.70-1.12)	0.97(0.72-1.30)	0.90(0.64-1.28)
Likely	1.60(1.33-1.93)	1.07(0.84-1.37)	1.01(0.77-1.34)	1.09(0.77-1.55)
Very likely	2.51(2.11-2.98)	1.28(1.00-1.66)	0.77(0.60-1.00)	0.91(0.64-1.29)
